# Effects of Personality Traits and Mentalization on Workplace Bullying Experiences among Intensive Care Unit Nurses

**DOI:** 10.1155/2023/5360734

**Published:** 2023-02-21

**Authors:** Sun Joo Jang, Eunhye Kim, Haeyoung Lee

**Affiliations:** ^1^Red Cross College of Nursing, Chung-Ang University, Seoul, Republic of Korea; ^2^Department of Nursing, Seoul National University Hospital, Seoul, Republic of Korea

## Abstract

**Aim:**

This study aimed to investigate the effects of intensive care unit nurses' personality traits and mentalization on workplace bullying after controlling for organizational culture.

**Background:**

Nurses' personality traits and mentalization may significantly influence workplace bullying.

**Methods:**

This cross-sectional study collected data from 416 nurses using an online survey in July 2022. Major variables were evaluated using the Pathological Narcissism Inventory, Perfectionistic Self-Presentation and Psychological Distress Scale, Short Dark Triad, Mentalization Scale, Positive Nursing Organizational Culture Measurement Tool, and the Negative Acts Questionnaire-Revised. . A multiple regression analysis was performed.

**Results:**

Narcissistic vulnerability, mentalization, and perfectionistic self-presentation significantly influence workplace bullying (victim aspect). Dark personality, mentalization, and perfectionistic self-presentation significantly influence workplace bullying (perpetrator aspect).

**Conclusions:**

Individual nurses could become either victims of bullying or perpetrators according to their personality traits. Therefore, it is necessary to determine their personality traits, so that they do not become influencing factors in workplace bullying. *Implications for Nursing Management*. Efforts at a nursing organization level are crucial to understand nurses' personality traits, enhance their mentalization, minimize the manifestations of the negative aspects of their traits, and positively affect the prevention and alleviation of workplace bullying.

## 1. Background

Workplace bullying causes practice errors among nurses, affects patient outcomes [1], significantly influences turnover [2], and increases workload and stress among the remaining nurses [3], resulting in a vicious cycle. Workplace bullying in nursing organizations remains an unresolved problem, and the understanding of contributing factors is still lacking [4]. In the nursing profession, research on workplace bullying has been conducted for the past 30 years, and various intervention methods have been suggested, but it is difficult to find noticeable improvements [5]. A systematic review of the literature on interventions over the past decade [6] also indicated that while it is significant that diversified antibullying interventions have been applied and evaluated, problems with conceptual clarity and effectiveness measurement still exist.

Meanwhile, the intensive care unit involves complex treatment and nursing due to higher patient severity than in other hospital departments; moreover, tasks must be performed quickly and accurately [7]. Furthermore, as most intensive care units are restricted, contact with outsiders such as patients and guardians is limited compared to general wards; however, there is much contact with medical personnel, resulting in conflicts [8]. In particular, the violence among intensive care unit nurses is mostly horizontal violence related to nursing work, that is, workplace bullying [9]. ICU nurses reported experiencing more work-related bullying than other types [10]. In a recent study of ICU nurses, the perpetrators of bullying were mainly nurses [11]. Workplace bullying among nurses is repeated, continuous, covert [4], and perpetrated by individuals [12]. Parke et al. [11] emphasized that negative behaviors such as bullying need to be recognized, reported, and effectively addressed rather than normalized within healthcare professions and workplace environments. Thus, nurses must be aware of their personality traits [13]. Understanding the relationship between personality traits and bullying may be a way to break the vicious cycle of bullying.

Johnson [14] recommended a multifaceted ecological model approach to understand bullying and develop interventions, wherein the microsystem is the smallest of the four interrelated systems, and the individual characteristics of the bully and target constitute an approach to this microsystem [14]. Personality traits refer to the patterns of thinking, feeling, and acting that have been formed over time, become stabilized under various conditions, and are distinguished from other characteristics [15]. Particularly, self and interpersonal functioning are key components of personality traits (American Psychiatric Association [16]); evaluating these factors is crucial to understanding personality pathology [17]. However, nurses' personality traits were analyzed as predictors of engagement among nursing professionals [18] and related factors of critical care nursing competence [19]. While previous studies on the relationship between personality and bullying exist, most have focused on adolescent bullying [20, 21]. A recent study targeting nurses [22] dealt with workplace bullying and personality traits as factors influencing nurses' health status but did not deal with the detailed characteristics of personality traits. To determine the relationship between workplace bullying and personality, this study focuses on pathological narcissism, perfectionistic self-presentation, dark personality, and mentalization among personality traits.

Pathological narcissism is characterized by a marked lack of empathy, a sense of entitlement, exploitative or arrogant behaviors or attitudes (narcissistic grandiosity), a desire for admiration, and frustration at the lack of admiration from other people (narcissistic vulnerability) [16]. While nurses with narcissistic personalities work with other nurses, such personality traits are likely to manifest in the form of workplace bullying victims or perpetrators. Previous studies show that narcissistic personalities induce counterproductive work behaviors toward colleagues [23] and that narcissistic behavior among nurses might have detrimental effects on their colleagues and institutions [24].

Perfectionist self-presentation refers to the desire to be seen as perfect [25]. People with high levels of perfectionistic self-presentation tend to promote behaviors perceived as perfect and conceal behaviors that they think are perceived as imperfect [26]. This means that beyond the desire to be perfect, these people are concerned with expressive desires, such as how they behave to be seen as perfect, which is an extreme and deceptive presentation of themselves to other people [26]. According to the Perfectionism Social Disconnection Model [27], perfectionistic self-presentation represents a set of defensive interpersonal behaviors including securing other peoples' love and respect (perfectionistic self-promotion) and preventing humiliation and rejection by other people (nondisplay of imperfection and nondisclosure of imperfection). Such behaviors have reportedly contributed to or exacerbated various forms of psychological distress and maladaptation, including depression, loneliness, hopelessness, and social anxiety [25, 28].

Paulhus and Williams [29] referred to individual tendencies to commit crimes or cause social problems as the “Dark Triad of personality,” which comprises three constructs: narcissism, Machiavellianism, and psychopathy; all three constructs commonly have negative characteristics such as coldness, lack of empathy, and duplicity [29]. Coldness and lack of empathy induce a tendency to manipulate other people [30]. Cold people appear attractive in short-term interactions with other people. However, this may negatively affect their relationships with others in long-term interactions owing to their low empathy and exploitative behaviors in pursuing their interests [31]. Disruptive behaviors (e.g., unproductive work behavior and abusive supervision) associated with the dark triad can adversely affect safety and productivity in organizations [32, 33]. Since a “dark” personality can be more excessively exhibited in a psychologically competitive organizational environment [34], it is important to identify the dark triad in employee selection and organizational culture improvement.

Mentalization refers to the ability to consider one's own and other people's mental states [35]. The ability to mentalize emotions is a construct of emotional competence in a broader sense and promotes an understanding of interpersonal relationship behaviors and self-regulation [36]. The deterioration of the ability to mentalize can put various psychological issues and bullies at risk. Moreover, victims are particularly vulnerable to mentalizing anger and are consequently more likely to perceive anger and happiness as behavioral conditions rather than mental states [37]. According to a previous study, the inability to mentalize can affect the ability to suppress violent behaviors [38].

A previous study on the effects of personality traits and perceived nursing organizational culture on workplace bullying among nurses [39] reported that higher perceived relationship-oriented and innovation-oriented cultures among nurses were associated with lower perceived workplace bullying, while higher nurse-perceived hierarchy-oriented and task-oriented cultures among nurses were associated with higher perceived workplace bullying. Other previous studies also reported that organizational culture was a significant influencing factor in workplace bullying [39, 40]. Therefore, this study aimed to analyze the effects of personality traits (pathological narcissism, perfectionistic self-presentation, and dark personality) and mentalization on workplace bullying among nurses after controlling for organizational culture.

## 2. Methods

### 2.1. Study Design

A cross-sectional study design was used.

### 2.2. Participants

The inclusion criteria were as follows: (1) nurses working in intensive care units (ICUs) at tertiary hospitals in South Korea at the start of this study and (2) nurses belonging to a nursing department. The exclusion criteria were as follows: (1) physician assistants, (2) nurses working in medical departments, (3) those not engaged in nursing-related work, and (4) those diagnosed with mental health problems or receiving treatment for mental health problems.

### 2.3. Data Collection and Ethical Considerations

An online survey was conducted in July 2022. The minimum number of samples required for multiple regression analysis was 322 with a two-tailed test, a significance level of 0.01, a power of 0.99, 16 predictors, and an effect size of *f*^2^ = 0.15 (medium) using the G*∗* power 3.1.9.7 version program. Considering a dropout rate of approximately 20%, 400 participants were targeted, and data were collected online through a Google Forms link in the form of an open call invitation. A warning was displayed to the participants not to respond more than once. The promotional notices on the survey were posted on online communities used mainly by nurses, such as tertiary hospital groupware bulletin boards and the online platform of the Korean nursing representatives. Finally, we collected 432 completed questionnaires; 416 (except for 16 that met the exclusion criteria) were finally analyzed.

This study was approved by the institutional review board (IRB) of the university to which the authors of this study belong (No. 1041078-202203-HR-96, approved on June 7, 2022). The participants who accessed the survey through the link received information about the study's purpose, methods, and voluntary withdrawal at any time. Those who agreed to participate in this study submitted electronic informed consent by clicking an “agree” button. All participants were provided with a guarantee of anonymity throughout the process.

### 2.4. Measurements

Information regarding participants' general (age, sex, marital status, religion, educational level, and subjective health status) and work-related characteristics (total working years, position, antibullying education, and experience of workplace bullying) was collected using self-report questionnaires. Specifically, regarding subjective health status, participants were asked, “How do you feel about your health condition?” Responses were provided using a 5-point Likert scale (1 = very bad, 2 = bad, 3 = moderate, 4 = good, and 5 = very good); a score of below three was considered to reflect a poor subjective health status, and a score of three or above was considered to reflect a good subjective health status. Participants were also asked to answer “yes or “no” to the question, “Do you attend religious services regularly?” to assess their religion status, referring to a previous study on nurses' workplace bullying [13]. Permissions were obtained for the original version/Korean version of all instruments.

#### 2.4.1. Pathological Narcissism

Pathological narcissism was evaluated using the Pathological Narcissism Inventory Korean version (a total of 35 items), which was culturally adapted for Koreans by Yang and Kwon [41], and the Pathological Narcissism Inventory (52 items), developed by Pincus et al. [42]. Additionally, this tool can be used to evaluate two phenotypes of pathological narcissism, namely, narcissistic grandiosity and narcissistic vulnerability. This tool is rated on a 7-point Likert scale (scoring range: 0–210 points); a higher score indicates more severe pathological narcissism. Cronbach's *α* at the time of the development of the original inventory [42], in the Korean version in a study by Yang and Kwon [41], and in this study was 0.95 (0.78–0.93), 0.92 (0.85–0.92), and 0.96 (0.91–0.95) respectively.

#### 2.4.2. Perfectionistic Self-Presentation

Perfectionistic self-presentation was evaluated using the Korean version [26] of the Perfectionistic Self-presentation and Psychological Distress scale (PSPDS) [25]. The PSPDS comprises three subdomains (perfectionistic self-promotion, nondisplay of imperfection, and nondisclosure of imperfection) with 27 items and is a 7-point Likert scale (1 = strongly disagree; 7 = strongly agree). Its Korean version comprises 19 items, of which 8 were deleted in cultural adaptation from the original scale. Cronbach's *α* during its development [25], in its Korean version [26], and this study was 0.91∼95, 0.85 (0.75∼0.88), and 0.90 (0.76∼0.91), respectively.

#### 2.4.3. Dark Personality

Dark personality was evaluated using the Korean version [43] of the Short Dark Triad (3 factors: narcissism, Machiavellianism, and psychopathy; a total of 27 items) [44]. The Korean version is a 17-item scale, in which 10 items from the original scale were deleted. Machiavellianism and psychopathy were grouped into one factor. Each item is rated on a 5-pointLikert-type scale (1 = strongly disagree; 5 = strongly agree); the total score ranges from 17 to 85 points. Cronbach's *α* at its development, in a Korean version in a study by [43], and in this study was 0.73∼0.78, 0.85 (0.75∼0.84), and 0.90 (0.80∼0.87), respectively.

#### 2.4.4. Mentalization

Mentalization was evaluated using the Korean version [45] of the Mentalization Scale (28 items) developed by Dimitrijević et al. [46]. It is a 25-item5-pointLikert-type scale (1 = completely incorrect; 5 = completely correct) comprising three domains: self-related mentalization, other-related mentalization, and motivation to mentalize. Cronbach's *α* of the original scale, the Korean version, and that in this study was 0.84 (0.74–0.79), 0.88 (0.74–0.84), and 0.84, respectively.

#### 2.4.5. Organizational Culture

Organizational culture was evaluated using the Positive Nursing Organizational Culture Measurement Tool [47]. This tool comprises four factors (positive leadership of the nursing unit manager, the pursuit of common values, forming an organizational relationship based on trust, and the fair management system), with 26 items. Each item is rated on a 5-pointLikert-type scale (scoring range: 24–120 points). A higher score indicates stronger perceived positivity toward nursing organizational culture. Cronbach's *α* of the tool during its development and in this study was 0.95 (0.83∼0.95) and 0.96, respectively.

#### 2.4.6. Workplace Bullying (Victim and Perpetrator Aspects)

The workplace bullying (victim aspect) was evaluated using the Korean version [48] of the Negative Acts Questionnaire-Revised (NAQ-R) [49]. This tool is a 22-item and 5-pointLikert-type scale (scoring range: 22–110 points). Cronbach's *α* of the original scale during its development [49] and in a Korean version in a study by Nam et al. [48] was 0.93 and 0.96, respectively. The workplace bullying (perpetrator aspect) was evaluated using the Negative Acts Questionnaire-Perpetrator [13], which was modified for the perpetrator aspect from the Negative Acts Questionnaire-Revised (NAQ-R) [49], and tested for its reliability and validity; Cronbach's *α* was 0.97 both during its development and in this study.

### 2.5. Statistical Analysis

Data were analyzed using the SPSS Statistics program 26.0 version (IBM Corp., Armonk, NY). Differences in workplace bullying according to participants' general and work-related characteristics were analyzed using a *t*-test and a one-way ANOVA. Ad hoc testing was further performed for variables with a significant intergroup difference using the Scheffé test. The correlations between pathologic narcissism (narcissistic grandiosity and narcissistic vulnerability), perfectionistic self-presentation, dark personality, organizational culture, mentalization, workplace bullying victim, and perpetrator aspects were analyzed using Pearson's correlation coefficients. Multiple regression analysis was also performed to identify the influencing factors of workplace bullying from the victim and perpetrator perspectives. The normality of the residuals was determined using the Kolmogorov–Smirnov test.

## 3. Results

### 3.1. General and Work-Related Characteristics of the Participants

The mean age and total working experience of the participants were 30.82 years old (standard deviation: 5.47 years) and 5.96 years (standard deviation: 4.49 years), respectively. Of the 416 participants, 380 (91.3%) were female, 299 (71.9%) were unmarried, 340 (81.7%) had a bachelor's degree, and 369 (88.7%) were staff nurses (Table 1).

### 3.2. Comparison of Workplace Bullying according to General and Work-Related Characteristics

The score for workplace bullying (victim aspect) was statistically significantly higher among women, those who attended religious services, staff nurses, and those with poor subjective health status. The score for workplace bullying (perpetrator aspect) was high among those with a religion, 3-year nursing college graduates, poor subjective health status, and those who completed antibullying education within 1 year (Table 2).

### 3.3. Correlation among Major Variables

Workplace bullying (victim aspect) was positively correlated with narcissistic grandiosity, narcissistic vulnerability, perfectionistic self-presentation, and dark personality, whereas it was negatively correlated with working years, mentalization, and organizational culture. Workplace bullying (perpetrator aspect) was positively correlated with working years, narcissistic grandiosity, narcissistic vulnerability, perfectionistic self-presentation, and dark personality, whereas it was negatively correlated with mentalization and organizational culture. Additionally, workplace bullying (victim aspect) and workplace bullying (perpetrator aspect) (Table 3) were positively correlated.

### 3.4. Factors Affecting Workplace Bullying

#### 3.4.1. Workplace Bullying (Victim Aspect)

Variables that significantly differed in workplace bullying (victim aspect) according to participants' characteristics and those that were significantly correlated with workplace bullying (victim aspect) were input into a regression model using the enter method. There was no multicollinearity between the independent variables (variance inflation factor: 1.04–3.62). Moreover, the Durbin–Watson index was 2.03. This confirmed the independence between error terms, thereby satisfying the assumptions of the regression analysis. The normality of the residuals was also confirmed (*Z* = 0.04, *p*=0.531). This regression model explained 36.2% of the workplace bullying variance. Narcissistic vulnerability (*β* = 0.25, *p* < 0.001), followed by organizational culture (*β* = −0.23, *p* < 0.001), mentalization (*β* = −0.16, *p* < 0.001), working years (*β* = −0.14, *p*=0.002), subjective health status (*β* = 0.12, *p*=0.005), and perfectionistic self-presentation (*β* = 0.11, *p*=0.019) were found to significantly influence workplace bullying (victim aspect). In other words, the score for workplace bullying (victim aspect) was higher among those who had a more severe narcissistic vulnerability, a more negative organizational culture, a lower mentalization, fewer working years, and a more severe perfectionistic self-presentation compared with their counterparts (Table 4).

#### 3.4.2. Workplace Bullying (Perpetrator Aspect)

Variables that significantly differed in workplace bullying (perpetrator aspect) according to participants' characteristics and those that were correlated with workplace bullying (perpetrator aspect) were input into the regression model using the enter method. There was no multicollinearity between the independent variables (variance inflation factor: 1.07–3.65). Moreover, the Durbin*–*Watson index was 1.90. This confirmed the independence between error terms, thereby satisfying the assumptions of the regression analysis. The normality of the residuals (*Z* = 0.05, *p*=0.269) was also confirmed. This regression model explained 41.0% of the workplace bullying (perpetrator aspect) variance. Dark personality (*β* = 0.48, *p* < 0.001), followed by mentalization (*β* = −0.17, *p* < 0.001), education level (*β* = 0.15, *p*=0.023), perfectionistic self-presentation (*β* = −0.13, *p*=0.007), and subjective health status (*β* = 0.13, *p*=0.001) were found to significantly influence workplace bullying (perpetrator aspect). Specifically, the score for workplace bullying (perpetrator aspect) was higher among those with a darker personality, lower mentalization, a weaker perfectionistic self-presentation, and poor health status and those who graduated from a three-year nursing college compared with their counterparts (Table 5).

## 4. Discussion

This study investigated the effects of personality traits and mentalization (individual factors) on workplace bullying among nurses after controlling for organizational culture (organizational factor). It demonstrated that narcissistic vulnerability had the greatest influence on workplace bullying (victim aspect) among nurses. People with more severe narcissistic vulnerability may experience more negative emotions, such as depression, owing to their unrealistic expectations; thus, they demand only positive responses from others [17] and may also feel ashamed for seeking recognition from others [50]. A previous study on the effects of personality factors on workplace bullying among nurses reported that narcissistic vulnerability was significantly positively correlated with depression, anger, and negative emotions and negatively correlated with positive emotions [41]. Therefore, nurses who exhibit narcissistic vulnerability are considered vulnerable to other peoples' negative evaluations or comments and are highly likely to perceive them as workplace bullying. Therefore, interventions such as emotion regulation and self-esteem regulation must be provided to nurses to actively regulate emotions and self-esteem according to their levels of narcissistic vulnerability [13, 41].

Another major influencing factor of workplace bullying (victim aspect) was mentalization. Mentalization is the ability to focus on and understand the mind of oneself or other people [35]. Low or impaired mentalization can cause difficulties in inferring the mental activity of oneself or other people, resulting in psychopathologies such as difficulties in emotion regulation and interpersonal problems [45]. Conversely, high mentalization has been associated with high life satisfaction, low depression, and low anxiety [46, 51]. Mentalization is developed through individuals' attachment relationships with their primary caregivers during their growth process but is also a fragile brain function that can be easily damaged by stressful situations [52]. Therefore, nurses with this tendency may be more likely to experience workplace bullying (victim aspect). It is thus necessary to develop and provide nurses with interventions that can reduce stress, help in coping with stress appropriately, and maintain stable mentalization.

Perfectionistic self-presentation also impacted workplace bullying (victim aspect). Specifically, higher scores for workplace bullying (victim aspect) were associated with higher perfectionistic self-presentation. People with high perfectionistic self-presentation conceal their imperfections and tend to appear perfect to consequently receive and maintain a favorable reputation from others [26]. As people with severe perfectionistic self-presentation tend to avoid other peoples' negative evaluations of themselves and gracing their shortcomings [17, 26], they are presumably more vulnerable to other peoples' negative evaluations or criticisms of their shortcomings that they may receive at work.

This study also showed that dark personalities had the greatest influence on workplace bullying (perpetrator aspect). Coldness (lack of empathy), one of the common characteristics of the three constructs of dark personality, inevitably induces a tendency to manipulate other people [30]. This may result in low empathy and exploitative behavior so that one pursues their interests in long-term interactions with others [31, 43]. This characteristic may be the basis for a major factor affecting workplace bullying (perpetrator aspect).

Mentalization was a major influencing factor of workplace bullying (perpetrator aspect). Specifically, lower mentalization was associated with higher scores for both workplace bullying victim and workplace bullying perpetrator aspects. Previous studies showed that mentalization was negatively correlated with borderline personality traits, depression, and anxiety [45]. Bullying victims are particularly vulnerable to mentalizing anger [37], and the inability to mentalize may affect the ability to suppress violent behavior [38]. Therefore, lower mentalization is presumably associated with higher workplace bullying.

Perfectionistic self-presentation was also a factor affecting workplace bullying (perpetrator aspect). This study showed that the score for workplace bullying (perpetrator aspect) was higher among those with weaker perfectionistic self-presentation, which contradicts results showing that the score for workplace bullying (victim aspect) was higher among those with stronger perfectionistic self-presentation. As people with high levels of perfectionistic self-presentation tend to be concerned about how they appear to other people to avoid making mistakes in social situations and tend to value harmony with other people [53], they are likely to have low scores for workplace bullying (perpetrator aspect). A previous study [54] showed that other-oriented and socially prescribed perfectionism showed unique relationships indicative of social disconnection and hostility, whereas self-oriented perfectionism showed unique relationships indicative of social connection. That is, since the perfectionism social disconnection model may not be applicable to all forms of perfectionism, it is necessary to confirm the association between perfectionism and bullying (victim and perpetrator aspects) through additional research [54, 55].

### 4.1. Limitations

This study had some limitations. First, as this is a cross-sectional study, it was difficult to determine the causal relationship between the major variables. Second, because this study involved ICU nurses at tertiary hospitals in South Korea, the possibility of selection bias (sampling bias) cannot be eliminated. Accordingly, generalizing the results of this study is limited. Therefore, longitudinal studies are needed to determine the causal relationship between personality traits and workplace bullying. Moreover, subsequent studies including background factors such as religion, education level, and subjective health status, all of which may affect workplace bullying, are also needed to strengthen the evidence. Additionally, it is necessary to develop and implement education and intervention programs that can reduce negative personality traits among nurses and improve mentalization and conduct studies on the effects of respective programs.

## 5. Conclusion

This study investigated the effects of personality traits and mentalization on workplace bullying among nurses. Under tense and unique working conditions, nurses occasionally become either workplace bullying victims or perpetrators. Accordingly, there is a need for continuous efforts at a nursing organization to understand nurses' personality traits, develop the mentalization that enables them to understand their own and other peoples' thoughts, and establish a positive organizational culture so that nurses can understand and support each other.

## 6. Implications for Nursing Management

This study demonstrated that personality traits might influence workplace bullying among nurses. However, assuming that certain nurses may be at risk of becoming workplace bullying perpetrators or victims owing to their personality traits, such individual nurses should not be stigmatized as victims or perpetrators. Nursing managers can help ICU nurses improve their self-awareness of personality traits and recognize the relationship between personality traits and bullying to prevent workplace bullying. It is necessary to develop and implement interventions to improve and reinforce mentalization that can help one properly perceive and interpret the thoughts and situations of oneself and others. Additionally, efforts at the nursing organization level are needed so that nurses' personality traits can harmonize with the culture of nursing organizations and generate positive effects. This will contribute to improving the quality of patient care and forming a positive organizational culture. Moreover, based on the study findings, nursing managers can raise awareness of personality traits and prepare interventions at the organizational level to prevent and cope with workplace bullying of ICU nurses.

## Figures and Tables

**Table 1:** General and work-related characteristics (*N* = 416).

Characteristics	Categories	*N* (%)	*M* (SD)
Age (years)			30.82 (5.47)
Sex	Female	380 (91.3)	
	Male	36 (8.7)	
Marital status	Single	299 (71.9)	
	Married	117 (28.1)	
Religious service attendance	No	227 (54.6)	
	Yes	189 (45.4)	
Educational level	3-year college	50 (12.0)	
	Bachelor's degree	340 (81.7)	
	≥master's degree	26 (6.3)	
Total working years			5.90 (4.49)
Position	Staff nurse	369 (88.7)	
	Charge nurse	47 (11.3)	
Subjective health status	Poor	67 (16.1)	
	Good	349 (83.9)	
Antibullying education	No	238 (57.2)	
	Yes	178 (42.8)	
Experience of workplace bullying (victim aspect)	No	319 (76.7)	
	Yes	97 (23.3)	

*M*, mean; SD, standard deviation.

**Table 2:** Comparison of workplace bullying by general and work-related characteristics (*N* = 416).

Characteristics	Categories	*N*	Workplace bullying (victim aspect)			Workplace bullying (perpetrator aspect)		
			*M* (SD)	*t*/*F*	*p*	*M* (SD)	*t*/*F*	*p*
Sex	Female	380	53.69 (19.04)	2.11	0.036	37.29 (16.49)	0.60	0.550
	Male	36	46.69 (18.89)			35.58 (15.64)		

Marital status	Single	299	54.17 (19.16)	1.86	0.064	36.88 (16.48)	0.53	0.596
	Married	117	50.31 (18.75)			37.83 (16.26)		

Religious service attendance	No	227	50.93 (18.99)	2.54	0.011	34.44 (14.75)	3.75	<0.001
	Yes	189	55.67 (18.96)			40.40 (17.70)		

Educational level	3-year college (a)	50	54.66 (16.22)	0.93	0.398	45.42 (19.44)	8.54	<0.001 (a) > (b)
	Bachelor's degree (b)	340	53.20 (19.37)			35.68 (14.97)		
	Master's degree (c)	26	48.50 (20.74)			40.42 (22.64)		

Position	Staff nurse	369	53.74 (19.10)	1.98	0.048	37.08 (16.44)	0.23	0.820
	≥ charge nurse	47	47.89 (18.54)			37.66 (16.28)		

Subjective health status	Poor	67	65.54 (17.91)	6.07	<0.001	46.94 (20.40)	4.46	<0.001
	Good	349	50.69 (18.41)			35.27 (14.83)		

Antibullying education	No	238	52.49 (19.38)	0.73	0.467	35.16 (15.55)	2.88	0.005
	Yes	178	53.87 (18.76)			39.80 (17.17)		

Experience of workplace bullying (victim aspect)	No	319	47.15 (16.37)	13.89	<0.001	33.98 (13.49)	7.62	<0.001
	Yes	97	72.59 (13.75)			47.57 (20.44)		

M, mean; SD, standard deviation.

**Table 3:** Correlation among variables (*N* = 416).

	Working years	Narcissistic grandiosity	Narcissistic vulnerability	Perfectionistic self-presentation	Dark personality	Mentalization	Organizational culture	Workplace bullying (victim aspect)	Workplace bullying (perpetrator aspect)
	*r*(*p*)	*r*(*p*)	*r*(*p*)	*r*(*p*)	*r*(*p*)	*r*(*p*)	*r*(*p*)	*r*(*p*)	*r*(*p*)
Working years	1								
Narcissistic grandiosity	−0.01 (0.841)	1							
Narcissistic vulnerability	0.04 (0.368)	0.77 (<0.001)	1						
Perfectionistic self-presentation	0.03 (0.565)	0.48 (<0.001)	0.53 (<0.001)	1					
Dark personality	0.03 (0.489)	0.71 (<0.001)	0.68 (<0.001)	0.48 (<0.001)	1				
Mentalization	−0.11 (0.030)	−0.07 (0.144)	−0.28 (<0.001)	0.05 (0.330)		1			
Organizational culture	−0.01 (0.920)	−0.03 (613)	−0.19 (<0.001)	−0.01 (0.821)	−0.15 (0.002)	0.24 (<0.001)	1		
Workplace bullying (victim aspect)	−0.11 (0.023)	0.35 (<0.001)	0.49 (<0.001)	0.29 (<0.001)	0.36 (<0.001)	−0.29 (<0.001)	−0.36 (<0.001)	1	
Workplace bullying (perpetrator aspect)	0.10 (0.036)	0.41 (<0.001)	0.47 (<0.001)	0.17 (0.001)	0.57 (<0.001)	−0.31 (<0.001)	−0.11 (0.023)	0.53 (<0.001)	1

**Table 4:** Factors influencing workplace bullying (victim aspect) (*N* = 416).

Variables	*B*	SE	*β*	*t*	*p*	VIF	95% confidence interval	
							Lower	Upper
Constant	73.65	8.77		8.40	<0.001		56.41	90.89
Sex^†^	−4.50	2.72	−0.07	1.66	0.098	1.04	−9.85	0.84
Religious service attendance^†^	0.96	1.56	0.03	0.62	0.538	1.07	−2.10	4.02
Position^†^	1.81	2.63	0.03	0.69	0.493	1.24	−3.37	6.99
Subjective health status^†^	6.17	2.18	0.12	2.84	0.005	1.14	1.90	10.45
Working years	−0.59	0.19	−0.14	3.17	0.002	1.26	−0.96	−0.23
Narcissistic grandiosity	0.01	0.10	−0.01	0.02	0.981	3.18	−0.20	0.19
Narcissistic vulnerability	0.25	0.07	0.25	3.39	0.001	3.62	0.11	0.40
Perfectionistic self-presentation	0.13	0.06	0.11	2.35	0.019	1.54	0.02	0.24
Dark personality	0.14	0.10	0.08	1.39	0.166	2.31	−0.06	0.34
Mentalization	−0.30	0.08	−0.16	3.71	<0.001	1.26	−0.45	−0.14
Organizational culture	−0.23	0.04	−0.23	5.34	<0.001	1.21	−0.32	−0.15
Adjusted *R*^2^ = 0.36, *F* = 22.41, *p* < 0.001 Durbin–Watson's *d* = 2.03 (du = 1.89, 4-du = 2.11), Kolmogorov–Smirnov test (*Z* = 0.04, *p*=0.531)								

^†^Dummy variable (reference). Sex (female), religious service attendance (no), position (charge nurse), subjective health status (good). SE, standard error; VIF, variance inflation factor.

**Table 5:** Factors influencing workplace bullying (perpetrator aspect) (*N* = 416).

Variables	*B*	SE	*β*	*t*	*p*	VIF	95% confidence interval	
							Lower	Upper
(Constant)	27.70	8.02		3.45	0.001		11.93	43.47
Religious service attendance^†^	2.23	1.29	0.07	1.73	0.084	1.07	−0.30	4.77
Education level^†^	7.57	3.31	0.15	2.28	0.023	3.03	1.05	14.08
Subjective health status^†^	5.89	1.80	0.13	3.28	0.001	1.14	2.36	9.43
Antibullying education^†^	4.04	2.45	0.11	1.65	0.100	3.06	−0.78	8.87
Working years	0.17	0.15	0.05	1.14	0.255	1.13	−0.12	0.45
Narcissistic grandiosity	0.01	0.08	0.01	0.07	0.948	3.20	−0.16	0.17
Narcissistic vulnerability	0.08	0.06	0.10	1.34	0.182	3.65	−0.04	0.21
Perfectionistic self-presentation	−0.12	0.05	−0.13	2.71	0.007	1.54	−0.22	−0.03
Dark personality	0.70	0.08	0.48	8.35	<0.001	2.33	0.53	0.86
Mentalization	−0.27	0.07	−0.17	4.03	<0.001	1.30	−0.40	−0.14
Organizational culture	−0.01	0.04	−0.01	−0.28	0.783	1.19	−0.08	0.06
Adjusted *R*^2^ = 0.41, *F* = 27.03, *p* < 0.001 Durbin–Watson's *d* = 1.90 (du = 1.89, 4-du = 2.11), Kolmogorov–Smirnov test (*Z* = 0.05, *p*=0.269)								

^†^Dummy variable (reference), religious service attendance (no); education level (above bachelor's degree); subjective health status (good); antibullying education (no); SE, standard error; VIF, variance inflation factor.

## Data Availability

The data presented in this study are available on request from the corresponding author and with permission of the Institutional Review Board of Chung-Ang University.
